# Long-term survival following radiofrequency ablation of lung metastases in an elderly patient with calcaneal osteosarcoma: A case report and review of the literature

**DOI:** 10.1097/MD.0000000000026681

**Published:** 2021-08-27

**Authors:** Hiroyuki Futani, Haruyuki Takaki, Tatsuo Sawai, Junichi Taniguchi, Yasukazu Kako, Yoshi-Hiro Ide, Koichiro Yamakado, Toshiya Tachibana

**Affiliations:** a Department of Orthopaedic Surgery, Hyogo College of Medicine, Nishinomiya, Hyogo, Japan; b Department of Radiology, Hyogo College of Medicine, Nishinomiya, Hyogo, Japan; c Department of Surgical Pathology, Hyogo College of Medicine, Nishinomiya, Hyogo, Japan.

**Keywords:** lung, metastasis, osteosarcoma, radiofrequency ablation

## Abstract

**Rationale:**

Recently, the number of osteosarcomas has been increasing in elderly patients due to human longevity. Lung metastases are the primary cause of death from osteosarcomas. Complete resection of lung metastases can prolong the survival. However, complete resection in elderly patients is often difficult due to high risk of operative complications. Computed tomography (CT) guided radiofrequency ablation (RFA) is a minimally invasive technique to destroy tumor nodules using heat. In this report, we present the first case older than 65 years applying RFA for lung metastases due to osteosarcoma.

**Patient concerns:**

A 74-year-old male presented with 1-year history of heel pain. A conventional high-grade osteosarcoma in his calcaneus was diagnosed. Below-knee amputation was performed. However, lung metastases were found in both lungs 1 year after amputation. CT-guided lung RFA was chosen since surgical intervention for lung metastases was abandoned because of tumor multiplicity and medical comorbidities. A total of 18 lung metastases were treated by CT-guided RFA. The most frequent complication was pneumothoraxes in 4 of 8 (50%) procedures and chest tube drainage was required in 2 of these (2 of 8 (25%) procedures).

**Diagnoses:**

Six lung metastases of osteosaroma were found in both lungs at 1 year after surgery.

**Interventions:**

CT-guided lung RFA was performed. A total of 18 lung metastases were treated in 8 lung RF procedures.

**Outcomes:**

The patient has been alive with disease for 5.5 years after the initial surgery.

**Lessons:**

CT-guided lung RFA is effective for elderly patients with osteosarcoma lung metastases in spite of discouragement of lung metastasectomy due to multiplicity of metastases and medical-comorbidities.

## 1 Introduction

Osteosarcoma is a high-grade malignant bone neoplasm that commonly occurs in the metaphysis of long bones in the second and third decade of life.^[1]^ Recently, the number of osteosarcomas has been increasing in elderly patients due to human longevity. Despite the advent of combined surgical and chemotherapeutic interventions, the prognosis of patients with lung metastases of osteosarcomas is still poor ^[2]^, especially in patients older than 65 years.^[3,4]^ Complete resection of lung metastases can improve life prognosis.^[5]^ However, metastasectomy in elderly patients is often performed with considerable hesitation due to lack of adequate respiratory reserve, high risk of operative complications, or other medical comorbidities.^[6]^

Radiofrequency ablation (RFA) is a technique to destroy tumor nodules using heat. The major advantage of RFA is a minimally invasive and relatively safe technique. Recently, a few papers reported this technique against lung metastases of osteosarcomas.^[7–9]^ However, the feasibility of this technique has not been reported for elderly patients having medical comorbidities.

Here, we describe an elderly patient with osteosarcoma in calcaneus, who metachoronusly developed multiple lung metastases. Subsequently, he has been surviving a relatively long period (5.5 years) by the use of computed tomography (CT)-guided lung RFA against his lung metastases.

## 2 Case presentation

**Figure 1:**
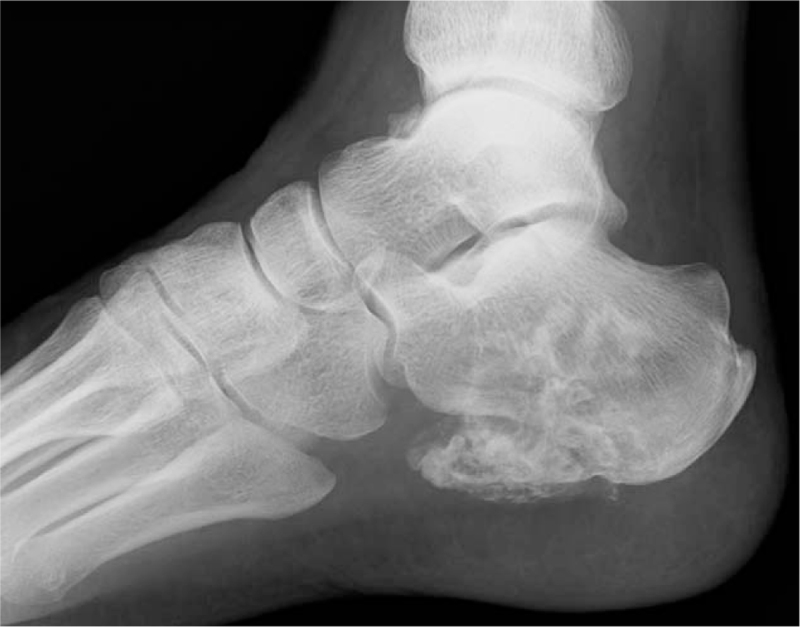
A lateral radiograph demonstrates radiolucency with an extraosseous mineralized mass in the calcaneal body.

**Figure 2:**
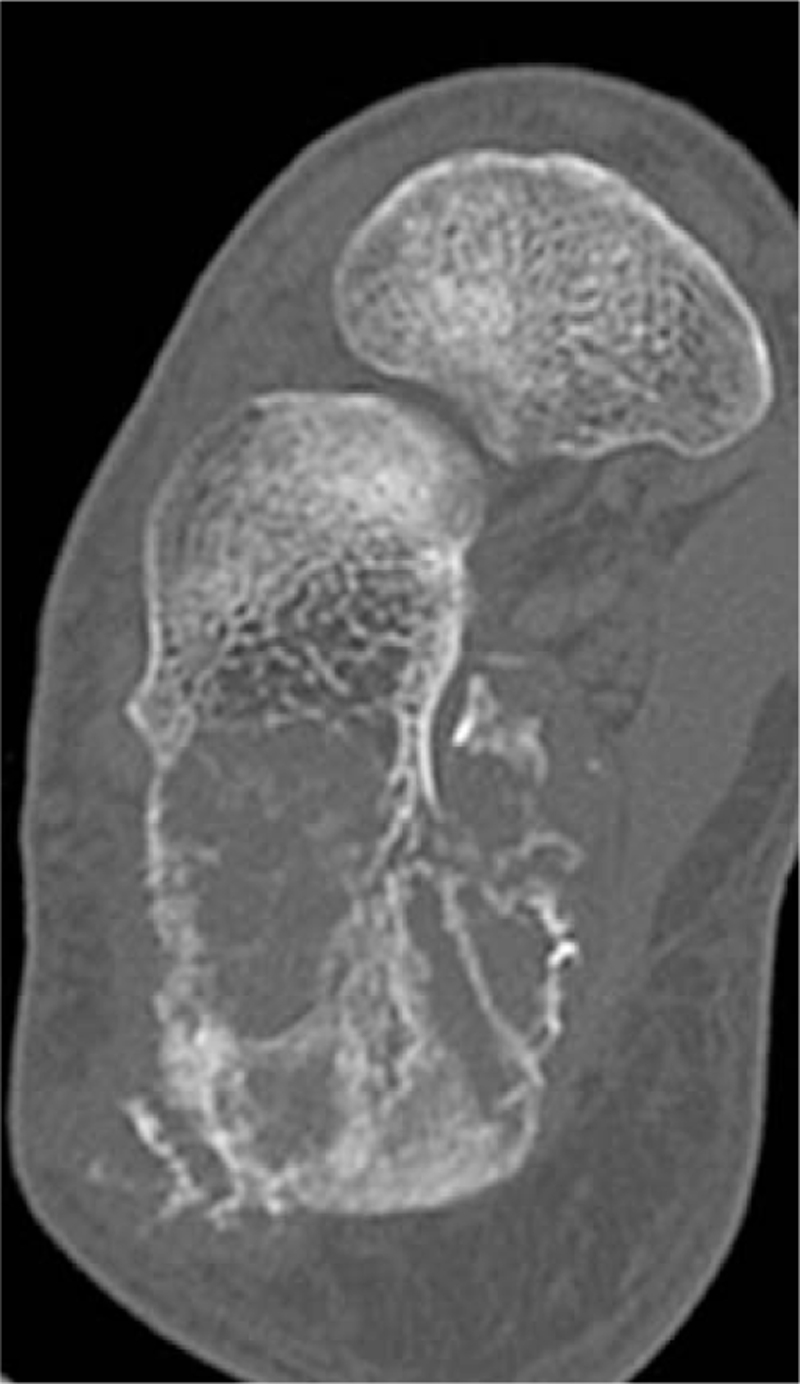
An axial CT image demonstrates osteolytic and ostosclerotic lesions with partial cortical destruction and fracture.

**Figure 3:**
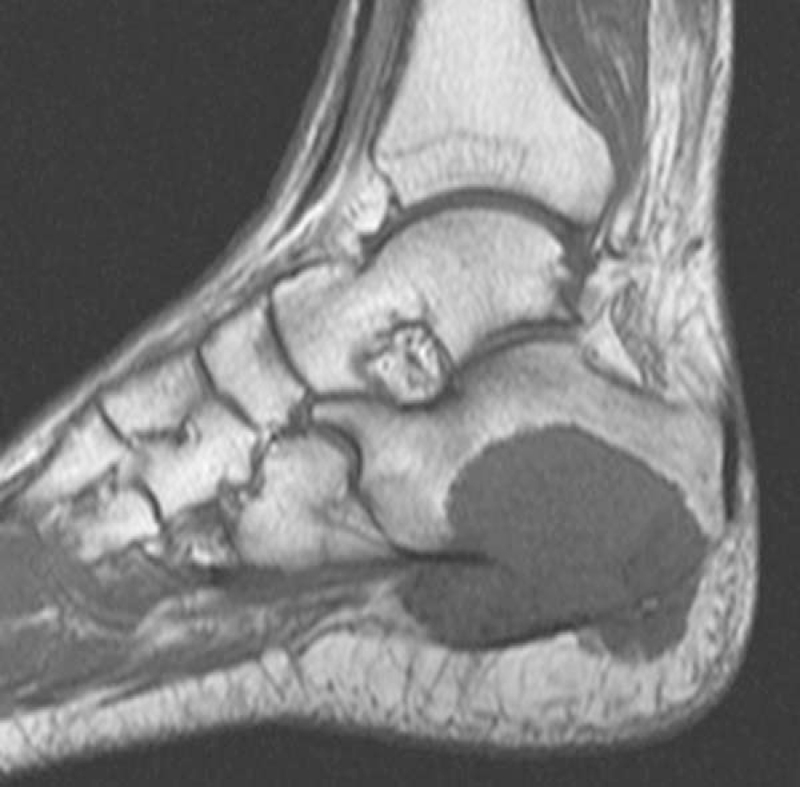
A sagittal T1-weighted MRI shows the area of decreased signal intensity area in the calcaneal body and the extraosseous mass. MRI = magnetic resonance imaging.

**Figure 4:**
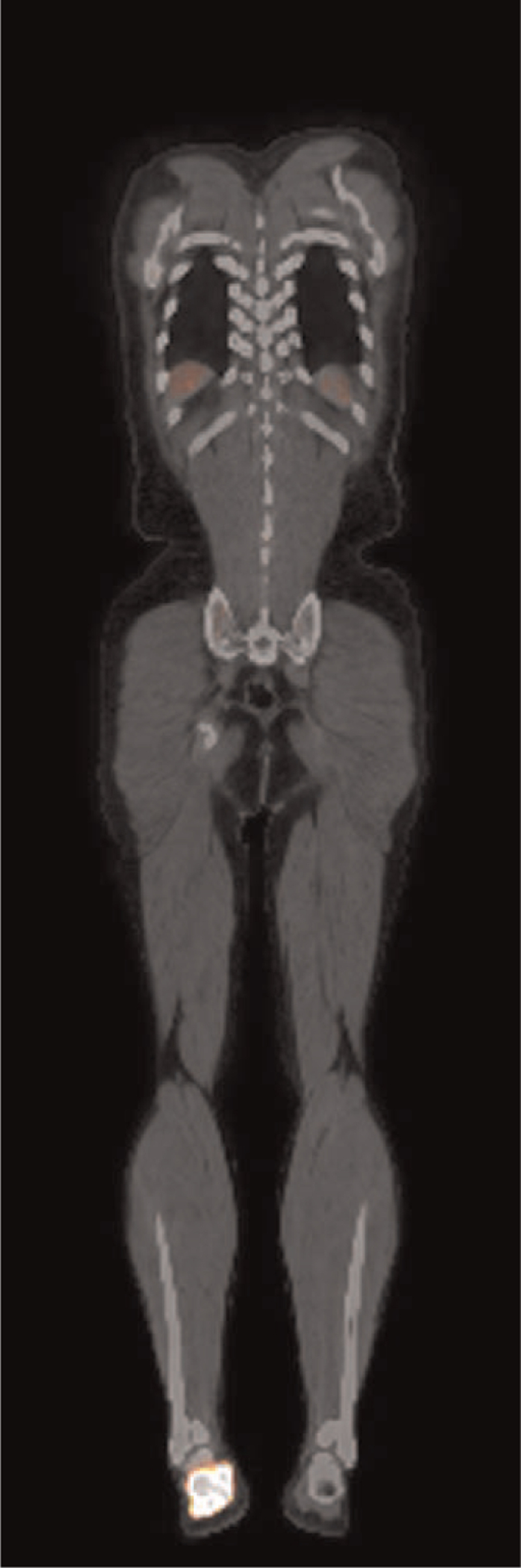
An anterior view of 18F-FDG PET images reveals an increased uptake in the calcaneus (SUVmas 6.6). No metastasis was found. PET = positron emission tomography.

A 74-year-old male presented with a right heel pain for 1 year. A tumor was found by radiography taken at a regional hospital when he noted increased heel pain. At presentation in our department, physical examination showed tenderness at the medial and lateral aspects of the calcaneal body. Radiography revealed an ill-defined osteolytic and osteoblastic lesion in the calcaneus with extension into soft tissues on the plantar aspect (Fig. 1). CT images clearly demonstrated an expanding osteolytic and osteoblastic lesion with extra-osseous lesions (Fig. 2). Magnetic resonance imaging revealed a 2.8 × 2.5 × 1.8 cm well-defined mass. The inferior cortical margin was destroyed with extra-osseous tumor. An area of low signal intensity was found by T1-weighted images (Fig. 3). On T2-weighted images, the tumor had a high signal intensity area including a low signal intensity area. These findings indicated a malignant tumor. Furthermore, positron emission tomography images clearly demonstrated an abnormal fluorodeoxyglucose F 18 (18F-FDG) uptake of the distal aspect of the left femur with maximum standardized uptake value of 14.8, indicating a malignant bone tumor (Fig. 4). No evidence of distant metastases was found.

**Figure 5:**
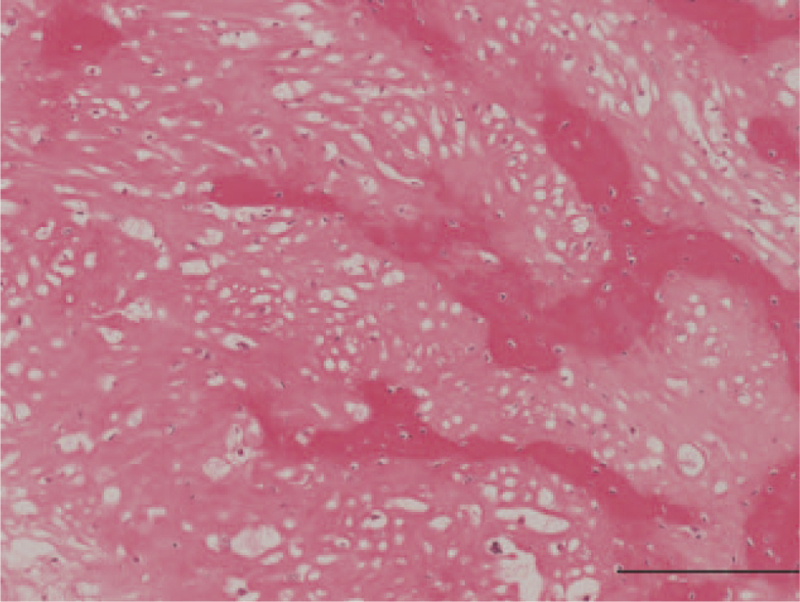
Histology of the biopsy specimen reveals malignant stromal cells forming osteoid (Bar=200 μm).

A needle biopsy was performed. The histology of the specimen showed osteoid matrix with malignant spindle cells, which was diagnosed as a conventional high-grade osteosarcoma (Fig. 5). Chemotherapy was not applicable since adverse effects were expected due to comorbidities of hypertension, diabetes mellitus, and renal dysfunction in addition to high age. A right below-knee amputation was performed to achieve a wide margin. Two months postoperatively, the patient regained the ability to walk with lower limb prosthesis.

**Figure 6:**
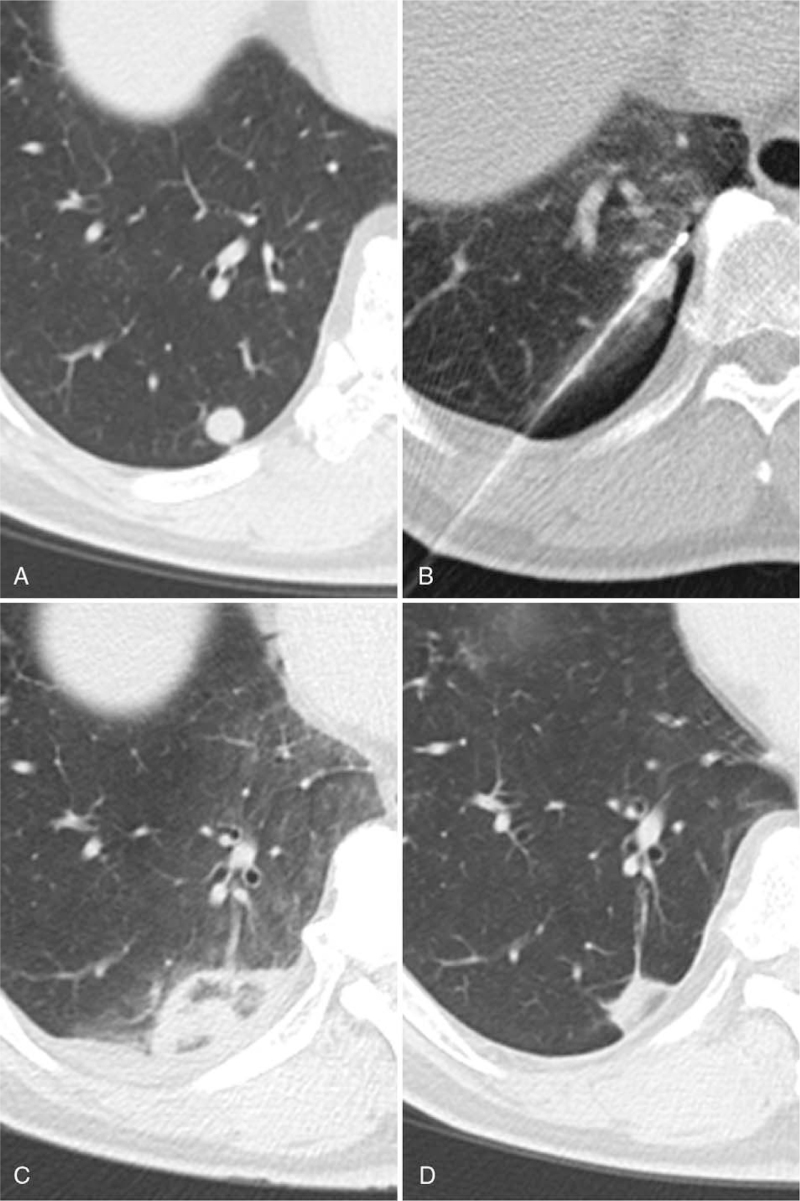
Sequential axial CT images before RFA (A), during RFA (B), at 3 days (C), 1 year (D) after RFA show dissolving of metastasis.

However, 6 lung metastases with a mean diameter of 6.3 ± 2.5 mm (range, 4–11 mm) were found in both lungs at 1 year after surgery. Surgical intervention for lung metastases was abandoned because of tumor multiplicity and high-risk surgery due to medial comorbidities. Therefore, lung RFA was chosen to treat lung metastases. Lung RFA was performed under moderate sedation and local anesthesia. Real-time CT fluoroscopy (SOMATOM, Siemens, Forchheim, Germany) was used to place the RF electrode in the tumors. An internally cooled electrode (Cool-Tip RF Ablation System, Covidien, Boulder, CO, USA) was used for the procedures. After the electrode was connected with the generator (Series CC, Covidien, Boulder, CO, USA), the RF energy was applied for 10 to 12 minutes at each site of the tumors using an impedance-control algorithm. The 6 lung metastases were treated by 2 RFA procedures, 3 in each procedure (Fig. 6).

Six months after initial lung RFA, new lung metastases developed. Therefore, additional lung RFA was performed. A total of 18 lung metastases with a mean diameter of 10.7 ± 6.8 mm (range, 4–25 mm) were treated in 8 lung RF procedures. The most frequent complication was pneumothoraxes in 4 of 8 (50%) procedures, and chest tube drainage was required in 2 of these (2 of 8 [25%] procedures). Mean duration of hospital stay for lung RFA was 5.3 ± 2.1 days (range, 3–10 days). The patient has been alive with disease for 5.5 years after the initial surgery.

## 3 Discussion

In a nationwide Japanese database from 2006 to 2013, 183 out of 1497 patients (22%) with osteosarcomas were found in the age of more than 65 years old.^[4]^ The need for studies on elderly patients with osteosarcomas is therefore increasing. Several papers have reported on poor prognosis of the elderly patients with osteosarcomas.^[3,4,10]^ In fact, Longhi et al ^[3]^ reported that 5 years over all survival was 33% for 29 patients with localized disease, however, 0% for 14 patients with lung metastases with high-grade osteosarcoma older than 65 years. Importantly, the present 74-year-old patient has been alive with disease for 5.5 years after the initial surgery.

Lung metastases are the primary cause of death from osteosarcomas. Complete surgical resections of all pulmonary lesions could lead to prolonged survival.^[5]^ However, surgical resection in elderly patients with osteosarcomas is not always applicable due to medical comorbidities. In fact, multiple surgical series have reported greater perioperative mortality in the elderly.^[11,12]^

Percutaneous CT-guided RFA has been reported to provide a safe and effective minimally invasive treatment for lung metastases.^[13]^ Lung RFA was introduced in 2000,^[14]^ and applied to patients with osteosarcoma in 2009.^[9]^ Thereafter, several authors have reported the feasibility of this technique in patients with lung metastases from osteosarcomas (Table 1). However, no paper has reported any patient older than 65 years with high-grade osteosarcoma and with lung metastases treated by percutaneous CT-guided lung RFA. In addition, the importance of the present case is the longest follow-up among patients with osteosarcomas treated by this technique (Table 1).

**Table 1:** RFA cases of lung metastases from osteosarcomas.

Authors	Year	Patient number	Age (years)	Treated nodules for each patient	Procedures for each patient	Pneumothorax	Tube drainage	Follow-up (months)
Hoffer et al^[9]^	2009	8	10–20	1–6	1–6	2 (9%)	1 (4%)	5–48
Saumet et al^[8]^	2015	10	6–22	1–4	1–2	3 (23%)	1 (8%)	15–51
Yevich et al^[7]^	2016	10	7–12	1–5	1–2	3 (27%)	1 (9%)	4–42
Present case	2021	1	74	18	8	4 (50%)	2 (25%)	66

In our institute, RFA is applied to tumor size of 3 cm or less. Basically, the electrode is placed in the center of the tumor when the tumor size is 2 cm or less. The risk of failure of RFA is in patients with large pulmonary lesions. When the tumor size is larger than 2 cm, the electrode is placed sequentially at 2 to 4 different sites in the tumor based on size and shape.^[13]^ A maximum of 3 lung tumors, developing in one lung, can be treated on the same day.

The advantage of RFA is that it allows ablation of tumors without major damage to the surrounding normal parenchyma. It has been demonstrated that RFA does not change lung function parameters and is possible even in patients with severe respiratory dysfunction.^[15]^ In addition, this technique can be performed percutaneously under moderate sedation and local anesthesia. It can be applied several times in patients with severe comorbidities or/and elderly patients. In the present case 8 lung RFA procedures could be performed against a total of 18 lung metastases.

The most frequent complication is pneumothorax requiring chest tube drainage, which occurs in approximately in 10% to 23% of the procedures.^[6–8]^ Nakamura et al^[6]^ reported the feasibly of RFA in elderly patients more than 65 years old with soft tissue and bone sarcomas. In their series, chest tube drainage was required in 15 out of 65 procedures (23%) in 12 patients, which was not statistically different from the results of sarcoma patients younger than 65 years. In the present case, pneumothorax occurred in 4 of 8 (50%) and chest tube drainage was needed in 2 of 8 (25%) lung RFA procedures. However, no serious clinical deterioration was found.

The advantages and disadvantages of RFA and surgical resection for patients with sarcoma lung metastases are summarized in Table 2. RF ablation could be as effective as surgery. However, further prospective randomized controlled studies are needed to evaluate the efficacy of lung RFA for patients with osteosarcoma lung metastases by comparing with metastasectomy.

**Table 2:** Characteristics of RFA and surgical resection for sarcoma lung metastasis.

	RFA	Surgical resection
Invasiveness	Low	High
Repeatability	High	Low
Tumor size	Preferably 3 cm or less	Any
Tumor location	Any, preferably central	Any, preferably peripheral
Local control ability	Medium-high^∗^	High
Treatability of lymph node metastasis	No	Yes
Treatability of vascular invasion	No	Yes

∗ Local control ability of lung RFA depends on tumor size and location.

In conclusion, percutaneous CT-guided lung RFA is a less invasive and safe technique for the elderly patient with lung metastases of osteosarcoma in spite of discouragement of lung metastasectomy due to multiplicity of metastases and medical-comorbidities.

## Author contributions

**Conceptualization:** Hiroyuki Futani, Haruyuki Takaki, Yoshi-Hiro Ide.

**Data curation:** Tatsuo Sawai, Yasukazu Kako.

**Funding acquisition:** Hiroyuki Futani.

**Investigation:** Haruyuki Takaki, Koichiro Yamakado.

**Supervision:** Junichi Taniguchi, Koichiro Yamakado, Toshiya Tachibana.

**Writing – original draft:** Hiroyuki Futani, Haruyuki Takaki.
